# The Silent Victims: How the Israel-Palestine War Impacts the Management of Chronic Kidney Disease and End-Stage Renal Disease Patients

**DOI:** 10.7759/cureus.55488

**Published:** 2024-03-04

**Authors:** Abhi C Lohana, Amit Gulati, Jatender Kumar, FNU Shivani, Danesh Kumar

**Affiliations:** 1 Department of Internal Medicine, West Virginia University/Camden Clark Medical Center, Parkersburg, USA; 2 Department of Cardiology, Icahn School of Medicine at Mount Sinai, New York, USA; 3 Department of Internal Medicine, Brookdale University Hospital Medical Center, New York, USA; 4 Department of Internal Medicine, Ascension Saint Joseph, Chicago, USA; 5 Department of Internal Medicine, Henry Ford Jackson Hospital, Jackson, USA

**Keywords:** end-stage renal disease, medical humanitarian, public health, health policy, chronic kidney disease

## Abstract

This article discusses the multifaceted impact of wars and armed conflicts on healthcare systems, with a focus on the Israel-Palestine war and its consequences for individuals with chronic kidney disease (CKD) and end-stage renal disease (ESRD). The war has severely disrupted healthcare infrastructure, leading to damage or destruction of hospitals and clinics, shortages in medical supplies and staff, and interruptions in the delivery of essential services. This disruption poses significant challenges for the management of chronic conditions such as CKD and ESRD, where patients rely on regular and specialized care. The article highlights the logistical challenges and health risks faced by these patients, including the interruption of dialysis treatment, shortages of medications, and the impact of displacement on continuity of care. It also addresses the psychological toll on patients, emphasizing the increased stress, anxiety, and depression that can exacerbate their condition. The need for international aid and humanitarian efforts to support CKD and ESRD patients in conflict zones is underscored, along with the importance of addressing the root causes of the conflict to ensure the well-being of vulnerable populations.

## Editorial

Wars and armed conflicts inflict profound and far-reaching consequences on societies, extending well beyond the visible battlegrounds [1]. While the physical injuries and deaths caused by war are often visible and reported, the impact of war on the management of ill patients and hospital treatment is less known and acknowledged.

One of the major challenges of war is the disruption of healthcare infrastructure and services. War can damage or destroy hospitals, clinics, laboratories, pharmacies, ambulances, and other medical facilities, equipment, and supplies. War can also disrupt the delivery of essential medicines, vaccines, blood products, and other medical commodities [2].

Another challenge of war is the increase of healthcare needs resulting in an increase of communicable diseases, such as cholera, typhoid, tuberculosis, malaria, HIV, and COVID-19, due to overcrowding, displacement, malnutrition, poor hygiene, lack of immunization, and reduced healthcare services [3]. War can also cause an increase in non-communicable diseases, such as diabetes, hypertension, cardiovascular diseases, cancer, and chronic kidney disease (CKD), due to stress, trauma, lack of medication, lack of monitoring, and lack of preventive care [3]. In short, the impact of wars on the management of ill patients is a multifaceted crisis that extends across various dimensions of healthcare.

The Israel-Palestine conflict, a longstanding and complex geopolitical struggle, has far-reaching implications that extend beyond the political arena. Amid the chaos and violence, the healthcare system, particularly the management of chronic conditions, faces severe challenges. This situation highlights the often-overlooked consequences of the Israel-Palestine war on individuals battling chronic kidney disease (CKD) and end-stage renal disease (ESRD), shedding light on the unique hurdles faced by this vulnerable demographic.

Amid the ongoing Israel-Palestine war, healthcare infrastructure has become a casualty, impacting the provision of essential medical services. Hospitals and clinics, critical for the treatment of chronic conditions such as CKD and ESRD, have either been directly targeted or indirectly affected by the chaos. The result is a compromised ability to deliver specialized care to patients in need. All hospitals in Gaza City and northern Gaza are facing a severe shortage of fuel and medical supplies due to the ongoing war. They are expected to completely go out of service soon.

For CKD and ESRD patients, the consequences are particularly dire. Most of the patients rely on different types of dialysis, and each treatment session needs a different timing and place to provide a successful treatment [4]. Regular and timely access to dialysis, a life-sustaining treatment for those with advanced kidney disease, becomes a logistical nightmare. Dialysis centers are often damaged or rendered inaccessible, leading to potential lapses in treatment. This disruption not only endangers the immediate health of patients but also contributes to the progression of kidney disease and the risk of associated complications.

This war has disrupted not only the physical infrastructure but also the pharmaceutical supply chain. CKD and ESRD patients rely heavily on a consistent and uninterrupted supply of medications to manage their conditions and prevent complications [5]. The ongoing turmoil in the region interferes with the transportation and distribution of these crucial medications, leading to shortages and uncertainties in the availability of essential drugs.

Immunosuppressive drugs, vital for those with kidney transplants, become particularly scarce, jeopardizing the success of these life-saving procedures. Additionally, medications to control blood pressure, a critical aspect of kidney disease management, may be in short supply, putting patients at an increased risk of cardiovascular events and accelerated kidney function decline [1]. A major example is the lack of basic anesthesia, which has forced surgeons in Gaza to perform operations without anesthesia.

The Israel-Palestine conflict forces many individuals, including CKD and ESRD patients, to flee their homes in search of safety. Displacement brings a host of challenges, disrupting the continuity of care essential for managing chronic conditions. Patients find themselves in unfamiliar environments with limited access to healthcare facilities, making it difficult to adhere to treatment plans and maintain regular medical checkups.

Transferring medical records and maintaining communication with healthcare providers become formidable tasks amid displacement. The lack of a comprehensive medical history complicates the ability of healthcare professionals to tailor treatments and interventions to the specific needs of CKD and ESRD patients, further exacerbating the challenges faced by this already vulnerable population.

Beyond the physical health implications, the Israel-Palestine conflict exacts a heavy toll on the mental and emotional well-being of CKD and ESRD patients. The constant threat of violence, loss of loved ones, and the experience of displacement contribute to heightened stress, anxiety, and depression among this demographic. The psychosocial impact is not to be underestimated, as studies have demonstrated the significant role these factors play in the progression of chronic diseases, including kidney disease [2].

Stress and anxiety can lead to elevated blood pressure, a key factor in the progression of kidney disease. Depression may result in poor adherence to medications and lifestyle modifications, further compromising the overall health of individuals with CKD and ESRD [2].

In the face of these challenges, international aid and humanitarian efforts emerge as crucial lifelines for CKD and ESRD patients in conflict zones. Organizations and healthcare professionals work tirelessly to provide medical supplies, essential medications, and healthcare services to those in need. However, these efforts are not without their challenges.

Restricted access to conflict zones, besieged cities, and resource limitations hinder the effectiveness of humanitarian initiatives. International collaboration and advocacy for the protection of healthcare facilities and medical supply chains become imperative to ensure a sustained and comprehensive response to the healthcare needs of CKD and ESRD patients.

In conclusion, the impact of the Israel-Palestine war on the management of CKD and ESRD patients is a multifaceted crisis. The conflict's pervasive disruptions to healthcare infrastructure, medication access, and continuity of care compound the already intricate challenges faced by these vulnerable patients. The psychological toll, coupled with the difficulties of displacement, paints a poignant picture of the silent casualties amid geopolitical strife.

Addressing these issues requires not only immediate humanitarian efforts to provide medical aid but also a broader commitment to resolving the underlying root causes of the war. Recognizing and prioritizing the healthcare needs of CKD and ESRD patients in the Israel-Palestine war zone is not just a medical imperative; it is a humanitarian imperative that speaks to the broader call for equity, compassion, and a commitment to the well-being of all individuals, even in the midst of turmoil.
